# Federated ontology-based queries over cancer data

**DOI:** 10.1186/1471-2105-13-S1-S9

**Published:** 2012-01-25

**Authors:** Alejandra González-Beltrán, Ben Tagger, Anthony Finkelstein

**Affiliations:** 1 Computational and Systems Medicine, University College London, Gower Street, London WC1E 6BT, UK; 2 Department of Computer Science, University College London, Gower Street, London WC1E 6BT, UK

## Abstract

**Background:**

Personalised medicine provides patients with treatments that are specific to their genetic profiles. It requires efficient data sharing of disparate data types across a variety of scientific disciplines, such as molecular biology, pathology, radiology and clinical practice. Personalised medicine aims to offer the safest and most effective therapeutic strategy based on the gene variations of each subject. In particular, this is valid in oncology, where knowledge about genetic mutations has already led to new therapies. Current molecular biology techniques (microarrays, proteomics, epigenetic technology and improved DNA sequencing technology) enable better characterisation of cancer tumours. The vast amounts of data, however, coupled with the use of different terms - or semantic heterogeneity - in each discipline makes the retrieval and integration of information difficult.

**Results:**

Existing software infrastructures for data-sharing in the cancer domain, such as caGrid, support access to distributed information. caGrid follows a service-oriented model-driven architecture. Each data source in caGrid is associated with metadata at increasing levels of abstraction, including syntactic, structural, reference and domain metadata. The domain metadata consists of ontology-based annotations associated with the structural information of each data source. However, caGrid's current querying functionality is given at the structural metadata level, without capitalising on the ontology-based annotations. This paper presents the design of and theoretical foundations for distributed ontology-based queries over cancer research data. Concept-based queries are reformulated to the target query language, where join conditions between multiple data sources are found by exploiting the semantic annotations. The system has been implemented, as a proof of concept, over the caGrid infrastructure. The approach is applicable to other model-driven architectures. A graphical user interface has been developed, supporting ontology-based queries over caGrid data sources. An extensive evaluation of the query reformulation technique is included.

**Conclusions:**

To support personalised medicine in oncology, it is crucial to retrieve and integrate molecular, pathology, radiology and clinical data in an efficient manner. The semantic heterogeneity of the data makes this a challenging task. Ontologies provide a formal framework to support querying and integration. This paper provides an ontology-based solution for querying distributed databases over service-oriented, model-driven infrastructures.

## Introduction and background

Personalised medicine provides patients with treatments that are specific to their genetic profiles. The aim is to offer the safest and most effective therapeutic strategy based on the gene variations of each subject. To that end, it is necessary to interact across a variety of scientific disciplines, such as molecular biology, pathology, radiology and clinical practice. Disparate data types from these domains need to be shared and integrated efficiently.

In particular, this is appropriate to oncology, where knowledge about genetic mutations has already led to new therapies. Current molecular biology techniques (microarrays, proteomics, epigenetic technology and improved DNA sequencing technology) enable better characterisation of cancer tumours. The vast amounts of data produced coupled with the use of different terms in each discipline - referred to as semantic heterogeneity- make the retrieval and integration of information difficult.

The UK National Cancer Research Institute (NCRI) and the US National Cancer Institute (NCI) have implemented programmes focusing on building and deploying software infrastructures to manage and analyse data generated from heterogenous data sources. These are the NCRI Informatics Initiative (NCRI II) [1] and the cancer Biomedical Informatics Grid^® ^(caBIG^® ^) [2] programme. The NCRI II has developed the ONcology Information eXchange (ONIX [3]) portal, enabling the discovery and searching of biomedical resources. The caBIG^® ^programme has developed the caGrid [4] computing infrastructure, and associated tools, supporting a collaborative information network for sharing cancer research data. caGrid deals with syntactic and semantic interoperability of the data resources in a service-oriented model-driven architecture. Each data source is represented as an information model [5] in the Unified Modeling Language (UML) [6], and it is exposed as a data service. Semantic interoperability is achieved by using a metadata registry, which maintains the information models annotated with concepts from a domain ontology, namely the NCI thesaurus (NCIt) [7]. The data services also expose a common query interface based on the caGrid query language (CQL). CQL enables to query the data services relying on their individual information models, i.e. the UML models. The query functionality provided in caGrid does not, however, take into account the existing semantic annotations based on NCIt. While the domain ontology is used as a global schema for the specification of data sources, the queries are not written in terms of the global schema but rather on the structure of the shared data resources.

In this paper, we provide an analysis of caGrid's support for data integration and its querying capabilities. We extend caGrid with additional services to support ontology-based queries over the cancer research data resources, taking advantage of the existing semantic annotations. The biomedical researchers, as the end-users of our system, can query the distributed data resources using queries based on the domain knowledge (expressed as concepts from the NCIt ontology). Thus, it is not a requirement to know the underlying models as for CQL, and the queries are reusable across resources.

Our approach assumes that all data sources have a corresponding information model with semantic annotations, where each element in the model (e.g. classes and properties) is associated with one or more concepts from a domain ontology. These concepts provide unambiguous meaning to the model's elements and could potentially belong to several ontologies. We assume there are service-oriented interfaces to access to the metadata registry, which stores the models and annotations, and the data sources. While any ontology could be use for the annotations, NCIt is the primary ontology in caGrid and all the information models are annotated with it [4]. Thus, for our implementation we consider NCIt exclusively. Our evaluation is based on data services from caGrid: we use data schemas and annotations available in the caGrid metadata registry.

Our system provides a customised transformation from the annotated information models to an ontological representation using the Web Ontology Language version 2 (OWL2) [8]. OWL is a recommendation from the World Wide Web Consortium (W3C). Based on the ontological representations of the data resources, we have designed and developed a query reformulation approach that converts concept-based queries into CQL, the query language supported by the caGrid infrastructure. This approach is general and could be used to support other target query languages, as the only step dependent on caGrid is the final one. This paper presents significant improvements over our previous work [9]. We have extended our earlier work to support federated queries over the caGrid infrastructure, where the selection of join conditions is provided by a semantic analysis of the distributed resources. We present an exhaustive performance evaluation of the query reformulation for single data resources. We also present a graphical user interface: the *Cancer ONtology QUErying SysTem *(*COnQueSt*). *COnQueSt *offers an ontology-based view of the caGrid data resources, allowing resource-browsing as well as identifying the concepts used therein. It also supports a query wizard to build ontology-based queries, allowing the user selection of the relevant data sources with respect to the concepts used in those queries.

### Data integration systems

Data integration refers to merging data from independent sources and providing access to them through a unified view [10]. There exist two common approaches for the integration of data: the data-warehouse approach and the federated database approach [11].

The warehouse approach collates the data from several resources, translates them and combines them into a single repository. Queries are executed over the aggregated data, rather than the distributed sources of data. Hence, distribution problems are avoided such as network bottlenecks, the unavailability of sources or slow response times, are avoided. Moreover, the execution of queries is very efficient and it is possible to apply optimisations over the aggregated data. Having the data in a single repository also permits added value in terms of validation and annotation. On the other hand, the data may become stale when the content or structure of data sources change [11]. Addition of new data sources requires an expensive process of translating its content into the repository [11].

The federated databases approach is composed of a *mediator*: a run-time component that reformulates queries written in a *global-schema *(or *mediated schema*) to queries on local schemas for each distributed data source. In contrast to the warehouse approach, federation ensures that the latest version of the data and structures is considered. Additionally, new databases can be added easily. The distributed nature of the infrastructure, however, compromises query performance [11].

In the federated approach, there are several ways to represent the mapping between the global schema and the set of local schemas for the data sources [10]. Each mapping associates a query written over the global schema with a query written over the local schema. These queries could be written in distinct languages. The two main methods are called Global-As-View (GAV) and Local-As-View (LAV) [12]. In GAV, each element in the global-schema is associated with a query over a local data source - i.e., each element in the global schema is characterised as a view over the data source. On the other hand, in LAV the global-schema is specified independently from the sources and each element of the data source is associated with a query over the global-schema, meaning that the local sources are characterised as a view over the global-schema.

Halevy [12] compares the two approaches from the point of view of query processing. In summary, query processing in GAV systems is generally based on a simple unfolding strategy, as the mappings identify the sources queries corresponding to elements in the global-schema [10]. But for LAV systems, query processing is more complex; it is not straightforward to determine how to use the sources to answer a query over the global-schema, as each source maintains only a partial view of the data [10].

### caBIG^® ^semantic infrastructure

caGrid, the computing middleware in caBIG^®^, is a Grid [13] extended to support data modelling and semantics [4]. It follows a service-oriented, model-driven architecture, with a number of core services and corresponding application programming interfaces (APIs). In this section, we present the caBIG^® ^semantic infrastructure as an analogy with the metadata hierarchy in [9,14] and analyse the infrastructure in terms of its capabilities as a data integration system.

**Figure 1:**
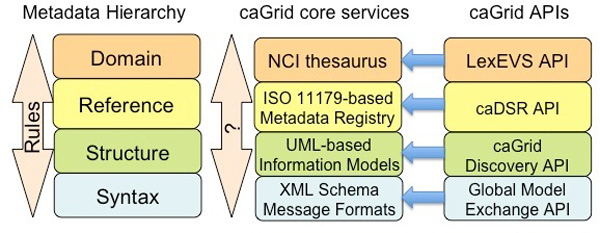
**caBIG^® ^semantic infrastructure core services**. **Figure 1**: caGrid core services, and their corresponding APIs, matched with the different levelsofthe metadata hierarchy. At the syntactic level, caGrid counts with XML Schemas to indicate the data types shared on the grid. These schemas are maintained in the Global Model Exchange, a service acting as an XML schema registry. The structural metadata is conformed by UML models, which can be accessed using the caGrid Discovery API. A metadata registry, based on the ISO/IEC 11179 standard, is used to manage common data elements (CDEs). The metadata registry, called caDSR, can be accessed with a specific API. A CDE is composed of an object class, a property and a value domain. These components correspond to a UML class, UML attribute and the attribute's data type, respectively, and each of them is associated with a set of concepts from an ontology. These mappings between structural elements and concepts constitute the reference metadata. The concepts are part of the domain metadata, and in caBIG^® ^mainly belong to the NCI thesaurus ontology. The LexEVS API allows to access the available terminologies.

caGrid follows a federated database approach, where each data source is autonomous and its owner is responsible for providing information about the resource. Each data source is exposed as a *data service*, using common interfaces and metadata at increasing levels of abstraction, including *syntactic*, *structural*, *reference *and *domain *metadata [14] (see Figure 1). Each data service is an object-oriented virtualisation of the underlying data [4]. The data types of the data source are available as eXtensible Markup Language (XML) schemas, managed by the Global Model Exchange (GME) service [4]. These schemas conform the syntactic metadata. The object-oriented representation of the data source is given as UML models, offering structural metadata about the data source. Each UML model is associated with semantic metadata, which indicates the meaning of the objects and associations between them. The semantic annotations come mainly from the NCIt ontology [7], which can be accessed via the LexEVS API [15]. NCIt is the primary terminology used in caBIG^® ^, but other well-structured ontologies should be suitable for the annotations. The NCI Enterprise Vocabulary Services (EVS) team reviews and approves suitable terminology for use in caGrid.

**Figure 2:**
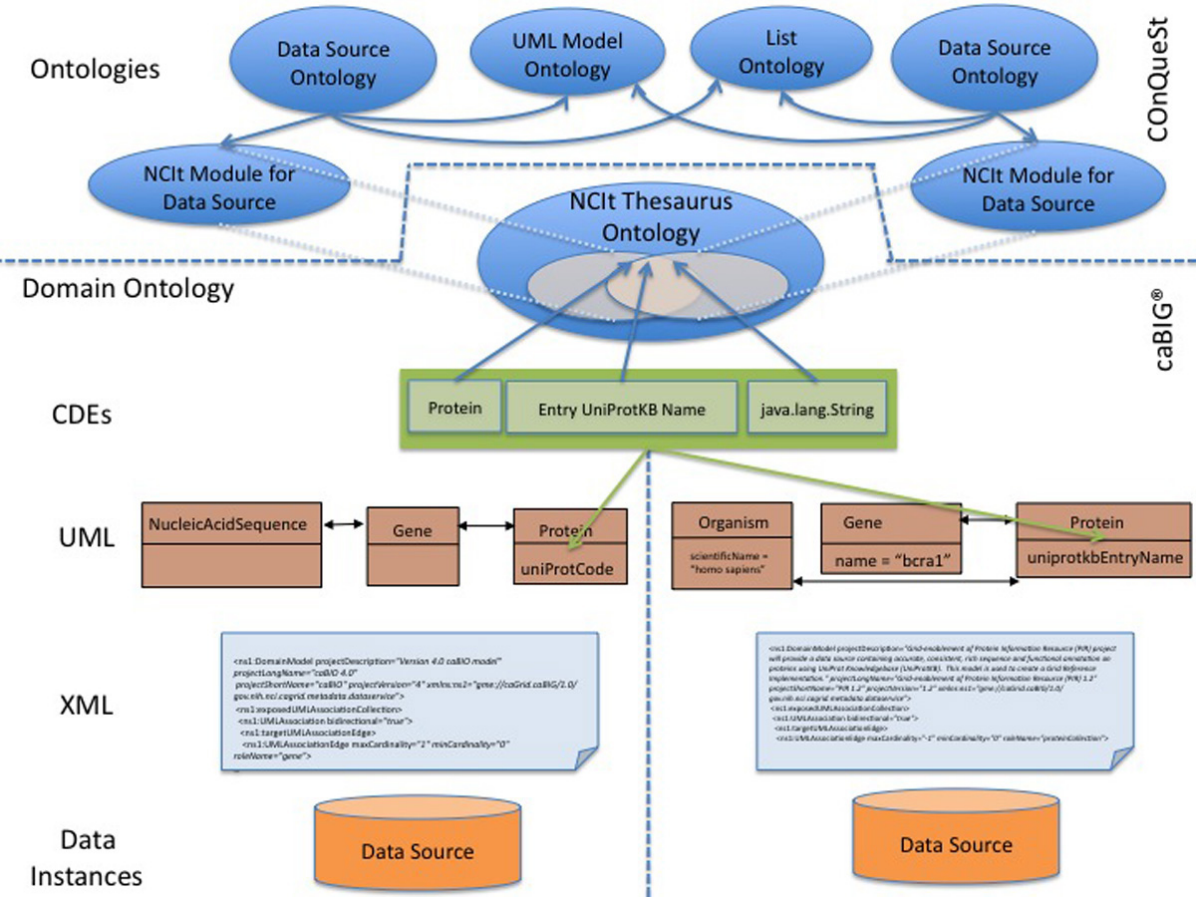
**caBIG^® ^semantic infrastructure and semantic layer built in our COnQueST system**. **Figure 2**: Different levels of metadata in the caBIG^® ^semantic infrastructure shown for two data sources that share a common data element (CDE). The CDE is annotated with concepts from the NCI thesaurus ontology. The top part of the diagram (above the dotted line) shows the ontologies built in the COnQueST system to facilitate ontology-based queries over caBIG^® ^data services.

The ontology-based annotations relate the domain concepts with the structural information of each data source, and constitute the domain metadata. The *cancer Data Standards Repository*, or *caDSR*, is a metadata registry based on the ISO/IEC 11179 standard [16]. caDSR manages common data elements (CDEs) and exposes them through the caDSR API. The CDEs provide the mappings between the ontology concepts (the domain metadata from the global schema) and the UML models for each available data service (the local schema). A CDE is composed of an *object class *that relates to a UML class, a *property *corresponding to a UML attribute, and a *value domain *corresponding to the data type of the attribute. The lower part of Figure 2 shows the different levels of metadata available in the caBIG^® ^semantic infrastructure. In caDSR, models are annotated with NCIt and we consider it as the only domain ontology for our implementation. As a data integration system, caGrid follows a federated approach with Local-As-View mappings, where the NCIt ontology offers a unified view of the resources. Each element of the data source (UML class, attribute and association) is related with a query (realised as a concept or set of concepts) over the global-schema (the NCIt ontology). In this way, the local sources are characterised as a view over the ontology. As seen before, CDEs offer these mappings and are maintained in caDSR.

As mentioned above, the data services expose access to the underlying data with a common interface based on the object-oriented (UML) model of the resource. This common interface also exposes a query processor based on the caGrid or Common Query Language (CQL) defined for caGrid. CQL is an object-oriented query language reflecting the underlying object model of the data resource while abstracting the physical representation of the data [4]. CQL allows the definition of one target object, representing the result of the query. Additionally, it is possible to add restrictions on associations or attributes for the classes intervening in the query. In this way, CQL is a navigational query language: it allows the navigation of the UML model through associations and the specification of conditions over the attributes of the classes traversed during that path navigation.

caGrid also supports basic distributed aggregations and joins of queries over multiple data services by means of the caGrid Federated Query Infrastructure [17]. The distributed queries are expressed in the distributed extension of CQL, called DCQL [18], which is translated into single resource queries in CQL. The service responsible for translating DCQL queries into CQL queries for the specific resources is the *Federated Query Processor *(FQP). Both CQL and DCQL are structural query languages, and require the user to know about the underlying object-oriented models of the resources.

The basic constructs for DCQL coincide with those of CQL, also permitting the navigation of the UML models through paths of UML associations and the specification of conditions on the UML attributes across the path. In addition, aggregated queries allow the same query to be run over more than one target service and return the combined results. Distributed joins, on the other hand, allow the definition of *foreign associations*. A *foreign association *element contains a *join condition element *and a *foreign object *element. The *join condition *element describes the logic for associating instances of the enclosing class with instances of the class in the remote data service that is described by the *foreign object *element.

To sum up, the caGrid infrastructure follows a LAV federated approach and maintains rich semantic metadata in caDSR. NCIt is the primary ontology in use, offering a unified view of the exposed data sources. However, the querying capabilities are not based in this global schema but on the object-oriented representation.

### Semantic web/linked data approach for querying and data integration

The *semantic web *was proposed as the method to solve the problem of semantic heterogeneity in the *World Wide Web *[19]. The proposal relies on extending the Web with a *semantic layer *that makes data not only human processable but also machine processable [20]. This approach allows the sharing and re-use of data on the Web, and it is sometimes called the *linked data *approach [21].

The semantic web methodologies, representation mechanisms and logics are largely based on database theory and practice [20]. However, there are important differences between database technologies and the semantic web - for example, databases are closed in nature (information not explicitly asserted is considered false) and their objects must be uniquely identified, while the semantic web assumes that information is incomplete and it recovers the notion of unique identifiers through Unique Resource Identifiers (URIs) [20].

The semantic web relies on a hierarchy of languages of increasing level of expressivity [20]. The Web Ontology Language (OWL) allows for the representation of classes and relations among them, which are organised in graph structures called *ontologies*. Each node represents a concept or class, and links codify logical relationships between the two concepts involved [20].

As discussed above, data integration depends on the mappings between component data schemas, or models, to a common schema. The semantic web supports the use of an ontology to integrate different databases [11,22]. In contrast to data models, ontologies encapsulate generic knowledge about a domain that can be reused across applications [11].

### Object-based queries

The concept of model-driven architectures (MDAs) [23], which was developed by the Object Management Group (OMG) [24], is based on platform-independent models and their transformations. The models document business functionality and behaviour of an application and are usually represented in UML. The models decouple the specification from the implementation that realises them, allowing for the independent evolution of the two. The models follow an object-oriented approach to software development, where the objects represent the entities in the system.

When database capabilities are combined with object-based virtualisation of software systems, the result is an object-oriented database management system. These systems offer query languages supporting the retrieval of objects stored in the system. The OMG proposed the Object Query Language (OQL), which is modelled after SQL, as a standard for object-oriented databases. As seen above, the caGrid infrastructure has developed its own object query language (CQL), based on the navigation of UML models [4]. While object-oriented databases provide powerful data abstractions, they generally lack a formal framework for query processing and query optimisation [25]. Fegaras and Maier [25] proposed the monoid comprehension calculus (MCC) as such formal framework. It is a calculus based on monoids and the homomorphisms between them. We use MCC for the query reformulation process described in the *Methods *section.

## Results and discussion

### CQL and DCQL analysis

A CQL query is defined by an XML document, which must comply to a given XML schema [26]. The schema indicates that a CQL query must specify a 〈Target〉 element, which is the data type of the query result. Optionally, an 〈Attribute〉 element might indicate a predicate over an attribute of the object with a 〈Target〉 type and an 〈Association〉 may specify a link with a related object. Next, we show how a CQL query is built recursively presenting it as a context-free grammar, where 〈CQLQuery〉 is the start symbol, ϵ is the empty string, 〈xsd:string〉 and 〈xsd:boolean〉 are the non-terminal variables representing the *xsd:string *and *xsd:string *data types, respectively. The CQL query context-free grammar is:

〈CQLQuery〉 → 〈Target〉 |

                         〈Target〉 〈 QueryModifier〉

〈Target〉 → 〈cqlObject〉

〈cqlObject〉 → 〈Name〉 |

                      〈Name〉 〈Attribute〉 |

                      〈Name〉 〈Association〉 |

                      〈Name〉 〈Group〉

〈Attribute〉 → 〈Name〉 〈Predicate〉 〈Value〉

〈Group〉 → 〈LogicalOp〉 〈Attribute〉 〈Group1〉 |

                 〈LogicalOp〉 → 〈Association〉 〈Group1〉

〈Group1〉 → 〈Attribute〉 〈Groupϵ〉 |

                   〈Association〉 〈Groupeϵ〉 |

                   〈Group〉 〈Groupeϵ〉

〈Groupe〉 → 〈Group〉|ϵ

〈Name〉 → 〈xsd:string〉

〈RoleName〉 → 〈xsd:string〉

〈LogicalOp〉 → **AND |OR**

〈Predicate〉 → **EQUAL_TO |NOT_EQUAL_TO **|

                      **LIKE |IS_NULL**|

                      **IS_NOT_NULL|LESS_THAN **|

                      **LESS_THAN_EQUAL_TO **|

                      **GREATER_THAN **|

                      **GREATER_THAN_EQUAL_TO**

〈Association〉 → 〈RoleName〉 〈cqlObject〉

〈Value〉 → 〈xsd:string〉

〈QueryModifier〉 → 〈countOnly〉 〈DistinctAttribute〉|

                             〈countOnly〉 〈DistinctAttribute〉 〈AttributeNames〉

〈countOnly〉 → 〈xsd:boolean〉

So, CQL traverses the UML class diagram graph, where the 〈Target〉 is the initial class, the 〈Association〉 conditions allow for path navigation by traversing sequences of consecutive classes and 〈Attribute〉 conditions apply locally to individual classes. The terminal symbols 〈Group〉 and 〈Group1〉 represent the combination of two or more constraints over a particular node in the UML class graph.

**Figure 3:**
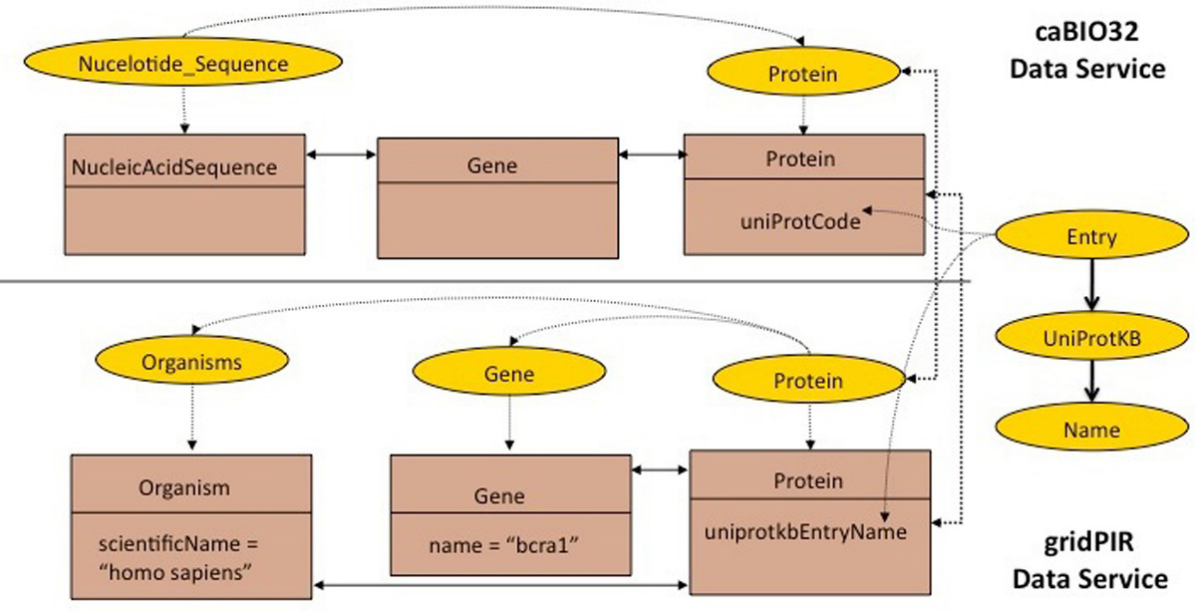
**DCQL Use Case**. Figure 3: Sections of the UML models of the caBIO and PIR data services showing the classes annotated with concepts included in the second query use case. This diagram corresponds to a solution of the query reformulation process involving multiple data services.

Now, we present an example from caBIO, where the CQLQuery encodes the traversal of the path from *NucleicAcidSequence *to *Protein *(see Figure 3).

〈CQLQuery〉 → 〈Target〉

                     → 〈qlObject〉

                     → 〈Name〉 〈Association〉

                     → 〈Name〉 〈RoleName〉 〈cqlObject〉

                     → 〈Name〉 〈RoleName〉 〈Name〉 〈Association〉

                     → 〈Name〉 〈RoleName〉 〈Name〉 〈RoleName〉 〈Name〉

                     → **NucleicAcidSequence geneCollection Gene proteinCollection Protein**

DCQL [18] is an extension of CQL to traverse two or more UML class graphs. The graphs are connected by the definition of join conditions, which determine how to associate instances of the enclosing class with instances of the class in the remote data service. DCQL specifies the service to which the query is sent to. This is a context-free grammar representing DCQL:

〈DCQLQuery〉 → 〈TargetObject) 〈targetServiceURL1〉

〈targetServiceURL1〉 → 〈targetServiceURL〉 |

                                    〈targetServiceURL〉 〈targetServiceURL1〉

〈targetServiceURL〉 → 〈xsd:string〉

〈TargetObject) → 〈dcqlObject〉

〈dcqlObject〉 → 〈Name〉

                        〈Name〉 〈Attribute〉 |

                        〈Name〉 〈dcqlAssociation〉 |

                        〈Name〉 〈ForeignAssociation〉 |

                        〈Name〉 〈dcqlGroup〉 |

〈dcqlAssociation〉 → 〈RoleName〉 〈dcqlObject〉

〈dcqlGroup〉 → 〈LogicalOp〉 〈Attribute) 〈dcqlGroup1〉 |

                        〈LogicalOp〉 〈Association〉 〈dcqlGroup1〉

〈dcqlGroup1〉 → 〈Attribute〉 〈dcqlGroupe〉 |

                          〈Association〉 〈dcqlGroupe〉 |

                          〈ForeignAssociation〉 〈dcqlGroupe〉 |

                          〈dcqlGroup〉 〈dcqlGroupe〉

〈dcqlGroupe〉 → 〈dcqlGroup〉 **| **ϵ

〈ForeignAssociation〉 → 〈JoinCondition〉 〈ForeignObject〉 〈targetServiceURL〉

〈JoinCondition〉 → 〈ForeignPredicate〉 〈localAttributeName〉 〈foreignAttributeName〉

〈ForeignPredicate〉 → **EQUAL_TO |NOT_EQUAL_TO |**

                                  **LESS_THAN **|

                                  **LESS_THAN_EQUAL_TO **|

                                  **GREATER_THAN **|

                                  **GREATER_THAN_EQUAL_TO**

〈localAttributeName〉 → 〈xsd:string〉

〈foreignAttributeName〉 → 〈xsd:string〉

Both CQL and DCQL are declarative, non-procedural languages.

### Ontology-based queries

We propose to exploit the caBIG^® ^semantic infrastructure as a data integration system following the LAV approach. This means that the NCI thesaurus ontology is considered as the global-schema and queries over the global-schema are reformulated as a set of queries over the data sources [10].

As a consequence, our system extends the caGrid querying functionality, which currently relies on the structure of the underlying data resources, i.e. their UML models. In caGrid, a biomedical researcher interested in retrieving data about, for example, a particular gene of interest needs to explore the UML model of each relevant data service and build a query considering the specific attributes and associations of the class maintaining the *Gene *objects. The queries can be built programmatically or also through the caGrid portal [27], which supports the exploration of the UML models and provides a query builder based on these models. The queries are specific for a data source and cannot be re-used.

On the other hand, users of our system can concentrate on the concepts from the domain, as represented by the NCIt ontology on cancer, and build the ontology-based queries which are *high-level *and *descriptive*. By a high-level query, we mean a query that can be written without specific details about the structure of the target resource. By a descriptive query, we refer to queries that provide the criteria for the desired data rather than the procedure to find the data. Thus, the ontology-based queries can be applicable to any of the underlying data resources, and our system reformulates them according to the specific UML models. The process is semi-automatic, in some cases requiring input from the users to select appropriate paths on the rewriting or join conditions, as will be explained in detail below.

Apart from the cancer concepts found in NCIt, the queries combine elements from an ontology we have built with metadata on UML models, namely the *UML model *ontology, and the list ontology [28], used to represent combinations of concepts that annotate elements from the data sources. The UML model ontology contains OWL classes to represent UML classes and attributes (*UMLClass*, *UMLAttribute*), OWL object properties to represent UML associations and the relationship between a UML class and its attributes (*hasAssociation*, *hasAttribute*) and a data property to represent the values of attributes (*hasValue*). The upper part of Figure 2 shows the ontologies built in our system in order to support ontology-based queries over the caBIG^® ^semantic infrastructure.

The navigational characteristics of the target object-query languages (CQL and DCQL for the caBIG^® ^infrastructure) are represented at the ontology level by the *hasAssociation *object property. Given two UML classes, they may have a direct UML association, or the association may arise by traversing an association path from the first class to the second one. In order for our system to deal with those paths of associations, without the user requiring knowledge of the specific underlying UML model, we define the *hasAssociation *property as transitive and use reasoning to determine the paths.

In the case of distributed queries, the semantic annotations of the models are leveraged to find the possible join conditions automatically. The join conditions are presented to the user, so that they can select the more biologically-relevant one, depending on the specific query.

#### Use cases

In this section, we present two simple but illustrative use cases, presenting a query for a single resource and a second query that requires the use of two resources to provide a result. The first use case will show how our system exploits the knowledge about the UML semantics. The second use case is based on the query presented in caBIG^® ^to demonstrate the federated query capability [29]. We will show the steps of our query reformulation process in the *Methods *section, giving examples based on these use cases. More than a thousand genetic mutations of the *BRCA1 *gene have been identified with increased risk of breast cancer in women [**?**]. The gene belongs to a class of genes identified as *tumour suppressors*, i.e. the protein that they produce helps prevent cells from growing and dividing too rapidly or in an uncontrolled way. The BRCA1 gene gives instructions for producing a protein that is directly involved in repairing damaged DNA. Additionally, the BRCA1 protein interacts with many other proteins, including other tumour suppressors and proteins that regulate cell division.

Some mutations on the *BRCA1 *gene can lead to the production of abnormally short versions of the BRCA1 protein. Other mutations may even prevent the protein being produced. Other mutations modify single amino acids in the resulting protein, or delete large segments of DNA from the BRCA1 gene.

As these mutations alter the normal function of the BRCA1 gene, their accummulatation can provoke uncontrolled cell division and growth, causing a tumour.

Taking into account this knowledge about the BRCA1 gene and knowing that its molecular location is at chromosome 17, a biomedical researcher investigating it will be interested in dealing with the results of the following queries:

Query 1

Find *single nucleotide polymorphisms *associated with the *chromosome *whose *name *is *17*.

Query 2

Find *nucleotide sequences *associated with the *gene *whose symbol is *BCRA1 *and whose *organism'*s *scientific name *is *homo sapiens*.

Using our system, these queries can be written using concepts from the NCI thesaurus ontology, whose correspondence with the above natural language phrases is straightforward. Our graphical user interface provides a *Query Builder *facilitating the query construction using concepts from NCIt. Once these queries are expressed with concepts, the internal representation is as follows (in Manchester OWL Syntax [30]):

Concept-Based Query 1

*Single_Nucleotide_Polymorphisms ***and hasAssociation some **(*Chromosome ***and hasAttribute some **(*Name ***and hasValue value ***"17"*)).

Concept-Based Query 2

*Nucleotide_Sequences ***and hasAssociation some **(*Gene ***and hasAttribute some **(*Gene_Symbol ***and hasValue value ***"BCRA1"*)) **and hasAssociation some **(*Organisms ***and hasAttribute some **(*Scientific*_*Name ***and hasValue value ***"homo sapiens"*)).

In order to answer these concept-based queries in the caBIG^® ^infrastructure, the researcher is able to find out through our interface about these two relevant data services:

• the cancer Bioinformatics Infrastructure Objects (caBIO) [31] data service: a robust resource for accessing molecular annotations from a variety of curated data sources, including CGAP, Unigene, the Cancer Gene Index (CGI) project ands the Pathway Interaction Database (PID);

• the Protein Information Resource (PIR) data service [32]: a data resource for genomic and proteomic information, which contains rich and high-quality annotated data on all protein sequences and is supported by the UniProt Knowledgebase (UniProtKB) and other relevant protein databases.

**Figure 4:**
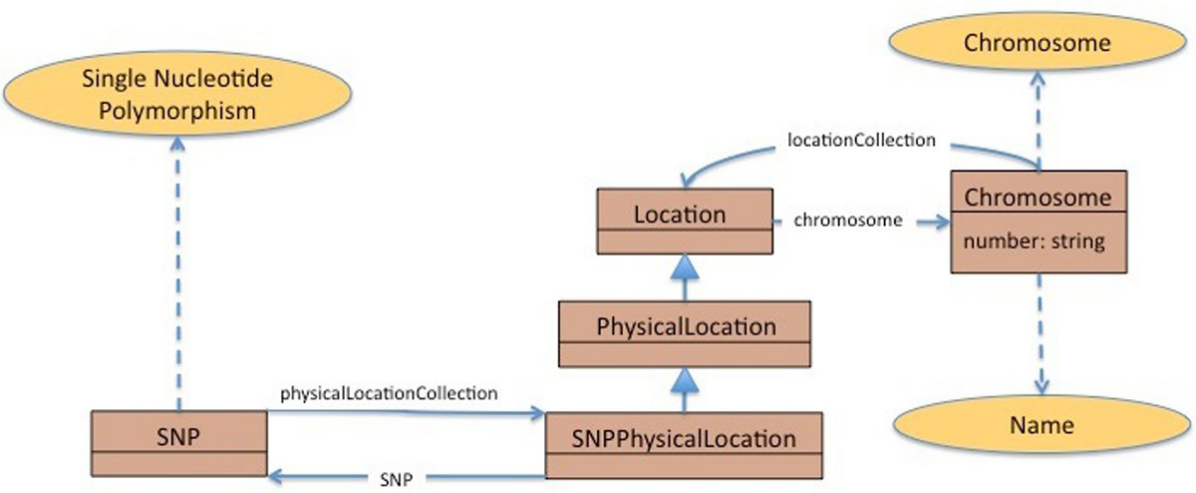
**Use Case**. **Figure 4**: Section of the caBIO UML model representing the relationship between the SNP class, corresponding to *single nucletoide polymorphisms *and the *Chromosome *class. This section of the UML model is relevant for the first query use case, where the solution involves a single target data service.

For the first query, the user chooses a single data resource as target, namely caBIO, as it contains data about single nucleotide polymorphisms and chromosomes. Figure 4 shows a section of the caBIO UML model corresponding to a possible path between the *SNP *class, corresponding to the concept *Single*_*Nucleotide*_*Polymorphism*, and the *Chromosome *class, corresponding to the homonym concept. We note that our system is able to reason about the structure of the data resource. Then, it automatically infers, based on the data service ontology, that the path between the two classes arises by considering the hierarchy of location classes (*SNPPhysicalLocation, PhysicalLocation *and *Location*) and that UML associations (in this case the *chromosome *association) are inherited by the sub-classes. The interpretations of the UML semantics are left to the user in the current caBIG^® ^infrastructure. Consequently, in caBIG^® ^there is the assumption that the user will be highly technologically knowledgeable.

For the second query, the user chooses the two data services as target, caBIO and PIR, in order to build a distributed query. While caBIO has data about nucleotide sequences and genes, PIR has information about organisms. Figure 3 shows sections of the two services' UML models, which refer to the classes annotated with concepts included in the concept-based query. Using our system, the researcher is presented with the possible join conditions for the distributed query. A join condition is composed of a pair of UML classes and a pair of UML attributes, corresponding to each of the classes. For the query to make sense, the join condition must contain semantically equivalent (or at least semantically related) classes and attributes. Two UML classes (attributes) are semantically equivalent if and only if they are annotated with the same concepts. By using a merged ontology combining the two data service ontologies, our system determines the list of possible join conditions. In this case, the join conditions include the pair of classes (*caBIO:Gene, PIR:Gene*) and (*caBIO:Protein, PIR:Protein*). Each pair of classes are annotated by the same concept, *ncit:Gene *and *ncit:Protein*. In turn, the semantically equivalent attributes for the pairs of classes are: (*caBIO:Gene*_*symbol, PIR:Gene*_*name*) and (*caBIO:Protein uniProtCode, PI:Protein*_*uniprotkbEntryName*). While the gene names (or symbols) are not unique, as there are several synonyms for each of the existing genes, the protein codes assigned by the UniProt Knowledge Base are unique. Thus, the biomedical researcher selects the *Protein *classes and codes from UniProt as a suitable join condition.

#### Software architecture

**Figure 5:**
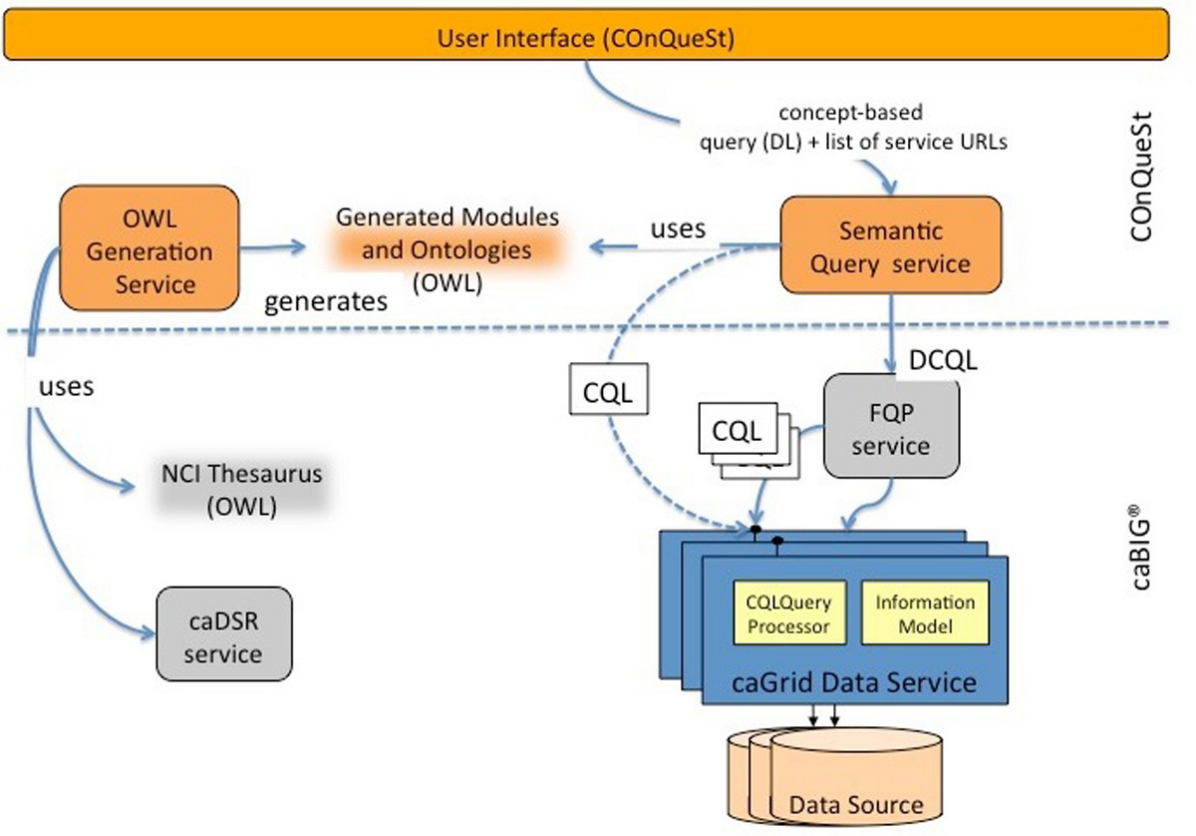
**System Architecture**. **Figure 5**: The caGrid service-oriented architecture (bottom part) extended with novel semantic services for the generation of ontologies and querying and a bespoke user interface (shown in the upper part of the diagram)

Figure 5 shows the extension of the caGrid service-oriented architecture with novel semantic services (shown in the upper part).

The semantic services are:

**OWL generation service**. This service generates NCIt modules for each of the available caGrid data services. The metadata is retrieved either from the *caDSR *service or directly from the individual data service. Additionally, this service generates OWL ontologies from the information models, i.e. the annotated UML models. The ontologies import the specific NCIt ontology module as well as the list ontology and the UML model ontology. The generated ontologies contain concepts and relationships but no data instances (or individuals).

**Semantic query service**. This service is responsible for rewriting, translating and processing semantic queries at different levels of abstraction, from ontology-based queries to a chosen target language. In the case of the caBIG^® ^infrastructure, the target languages are CQL or DCQL, depending on whether the query is applied to a single or multiple data sources, respectively. The approach utilises the Monoid Comprehension Calculus as an intermediate language, allowing the translation to different target languages for other infrastructures.

More details about these services are given in the *Methods *section.

### Implementation

We have implemented two modules, with the functionalities described above. The implementation was done in Java and uses caGrid version 1.3 [33], the OWLAPI version 3.1.0 [34] (after upgrading from OWLAPI version 2), and relies on the reasoners Pellet 2.2.2 [35] and HermiT 1.3.0 [36].

#### OWLGen caGrid analytical service

For the first module, we also produced a caGrid analytical service called the OWLGenService [37] and it is accessible through the *caGrid portal *[27].

The service provides a simple API allowing for:

• extraction of modules from NCIt

• data service ontology generation

Both methods accept a project short name and version from the *caDSR *service or the URL of the data service of interest.

#### COnQueSt graphical user interface

In order to demonstrate the functionality of the query rewriting process, we have developed a web-based interface, which we call *COnQueSt - Cancer Ontology Querying System*, that affords the user several key abilities;

**Browser **(inFigure 6) The user can browse the projects available in *CaDSR *and investigate the NCIt concepts in each project. We provide information such as definitions and links to the NCIm [38].

**Figure 6:**
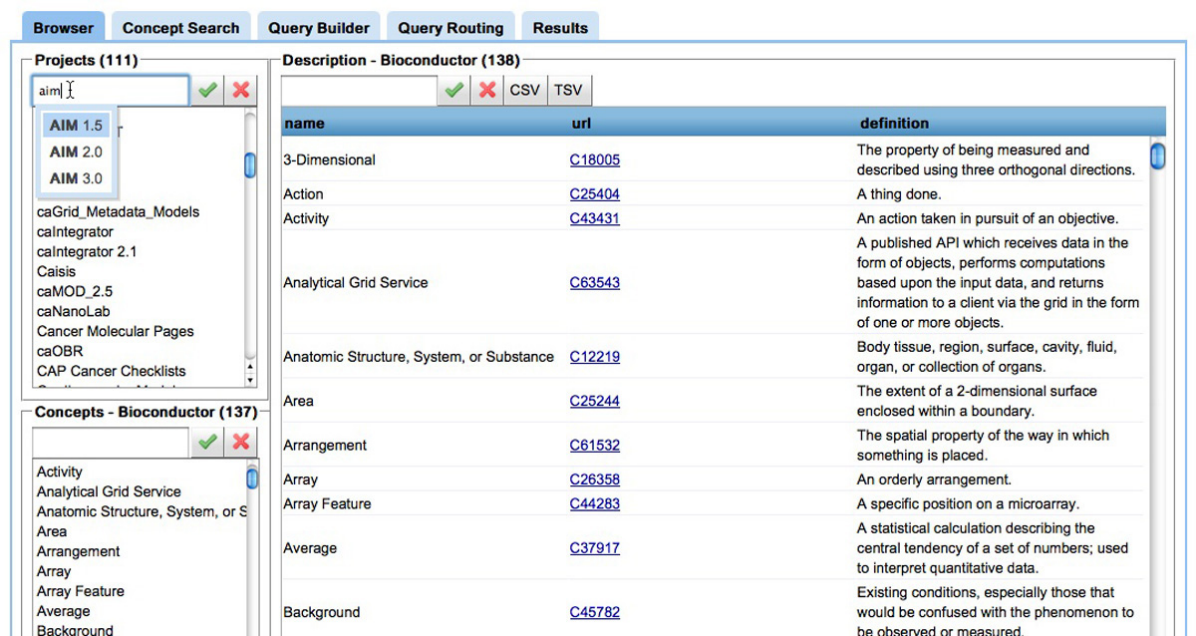
**Screenshot of the browser tool in COnQueSt interface**. **Figure 6**: The browser tool in COnQueSt interface: the upper left panel shows the list of projects (or information models) available, the bottom left panel shows the concepts used to annotate the selected project, and the right panel allows to view the concepts definitions, including links to the NCI thesaurus browser. All panels have a searching facility: for instance, it is possible to search projects by their name.

**Search Tool **(inFigure 7) The user can search for NCIt concepts, either by matching patterns or exact searches, returning metadata about the concepts and the projects that contain those concepts.

**Figure 7:**
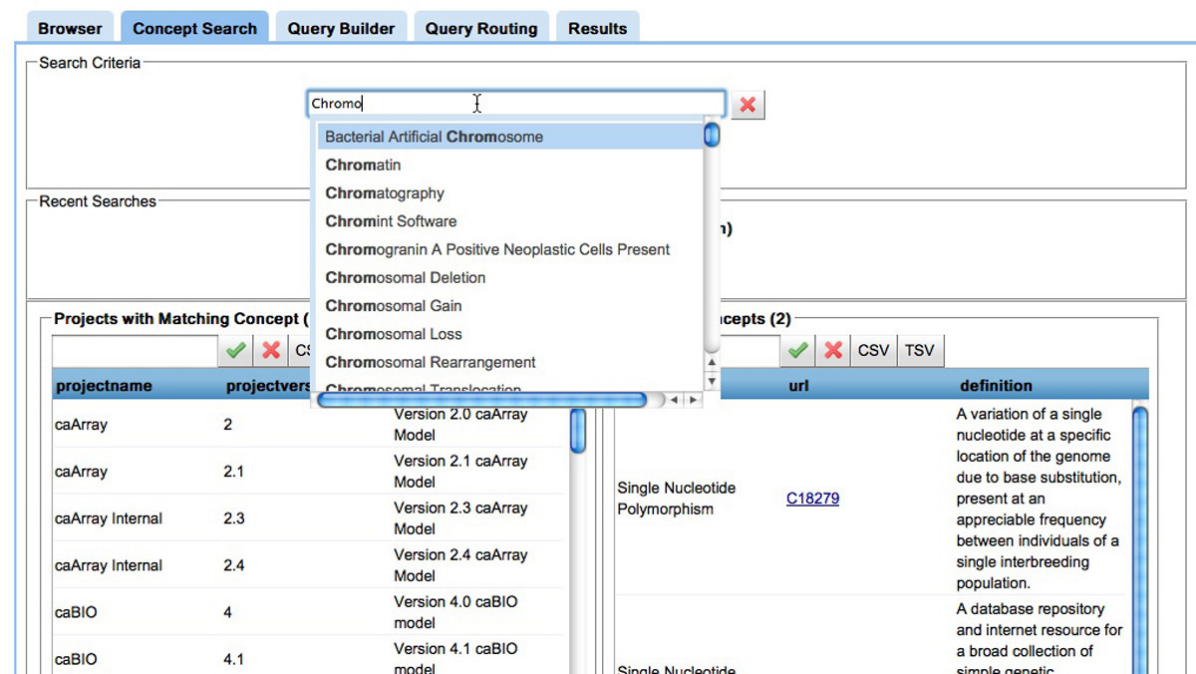
**Screenshot of the search tool in COnQueSt interface**. **Figure 7**: COnQueSt search tool: when searching for a concept, the result shown includes the projects (or information models) with matching concepts as well as the concepts themselves. While the "search" button considers all the concepts containing the search criteria, the "I'm feeling lucky" button retrieves the concept that matches exactly the search criteria.

**Query Builder **(inFigure 8) We provide a custom query-building interface that demands no prior knowledge of description logics or OWL class expressions. The query builder uses a point-and-click interface with auto-suggestion concept boxes that force the user to create syntactically valid, description-logic based queries.

**Figure 8:**
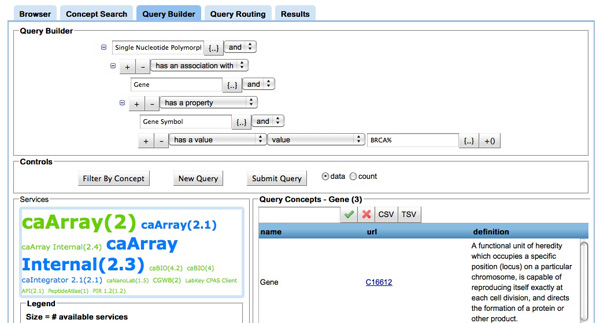
**Screenshot of the query builder in COnQueSt interface**. **Figure 8**: The COnQueSt query builder tool allows to search available concepts and to specify an association between them, to indicate that has a property specified by another concept or indicate a specific value.

**Query Rewriting **Users can interact with the query-rewriting process, choosing from the available UML extractions and selecting the appropriate paths during the path-finding stage. The user is prompted for a choice when required, the ultimate result of which is a CQL query that the user can inspect visually to verify the semantic correctness.

**Query Execution **(inFigure 9) Users can run the rewritten query against the service of their choice and retrieve and save their results in a variety of formats.

**Figure 9:**
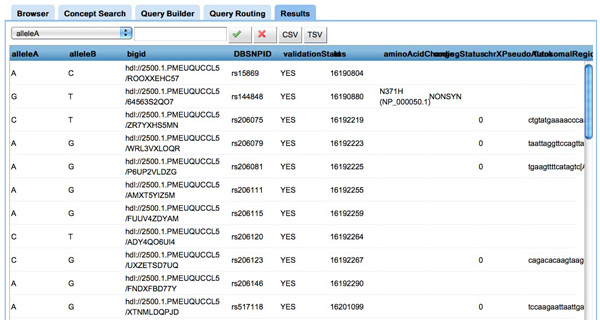
**Screenshot of the results panel in COnQueSt interface**. **Figure 9**: The query results panel shows a table listing the properties of each result object.

The interface has been developed using the Google Web Toolkit (GWT) with a MySQL Database backend. Client-server communications employ the Java RPC implementation^1^.

### Performance evaluation

For an evaluation of the query reformulation process, our experimental analysis covers the following:

1. We present some metrics to assess the OWL representation of the information models. In particular, since an important step in the rewriting process is the *property path finder*, we examine the sequences of concepts linked by object properties (paths).

2. We provide results of the generation times for the module extraction, the ontology generation and the inference of the ontologies using both the Pellet and HermiT reasoners. These results show that the generation of the ontologies, which enable our approach, can be done in a performant manner.

3. We perform an evaluation of the query rewriting process, showing a breakdown of the constituent parts of the rewriting algorithm.

4. We compare explanation generation times, simulating the request of 1-5 explanations, demonstrating the effects on the rewriting process.

The tests were run on a Red Hat Enterprise Linux Server release 5.3 (Tikanga 64 bit) and 48285 MB of RAM. The output files corresponding to the performance evaluation are available at [37].

### Analysis of the OWL representation of the information models

Throughout this section, we group caGrid projects into three distinct subsets: projects available from the *caDSR *service, data services that are registered with the *caGrid *default index service [39], and *Information Models *(those models that are supported by a deployed service from the *caGrid *Index Service). It should be noted that not all caDSR projects are included in the metrics; some contained errors (their semantic metadata is not complete or refers to an older version of the NCI thesaurus) and some models are targeted for data modelling, rather than specifically holding data, making them unrepresentative for our system. Out of the 136 projects in caDSR, 16 were excluded from the analysis for these reasons. However, none of the excluded projects had an associated service. Additionally, the *caGrid *subset has 63 services and *InfoModels *has 23 projects. The groups *caGrid *and *InfoModels *are the more relevant for our system, as it is only possible to execute CQL queries against projects that have an associated *caGrid *service. While *InfoModels *include a single project from caDSR for a set of deployed services corresponding to that project, caGrid may include the results for several services that correspond to a single model. Thus, the *caGrid *results will be skewed according to the relative weight of services as opposed to models.

There are several tools for establishing ontological metrics including ONTOMETRIC, OntoQA and Protégè as the main available proposals [40]. ONTOMETRIC [41] is a framework that allows users to measure the suitability of a particular ontology with respect to the requirements of their system. ONTOMETRIC provides a taxonomy of *characteristics *for each ontology, from which the user can choose a selection to compare against another ontology. While Proteégè is primarily a tool for creating and modifying ontologies, it does provide a limited selection of metrics for an ontology, but they are not semantic metrics. There are other ontology metrics that focus on cohesion, most of which focus on mining inconsistencies in the ontology [40]. While ontology metrics have been defined in several of these tools [40], these have focused on basic metrics (e.g. number of classes) or semantic-based metrics (e.g. relationship richness) that allow for the comparison and quality evaluation of the ontologies. Therefore, we will focus on the presentation of some bespoke metrics we developed to measure the proliferation and complexity of paths within the ontologies, as these will ensure the viability of our approach.

Our rewriting process seeks to remove the upper-level and transitive object property *hasAssociation *and express the query using only non-transitive properties, which correspond to the UML associations in the models. In order to achieve this, we consider the paths between pairs of concepts from the query connected through the *hasAssociation *property. The calculation of these paths is not trivial; there may be many intermediate nodes and there may be more than one path for a given pair of concepts. We define a *journey *as a traversal from one concept to another. A *journey *may have one or many paths, which represent the possible routes that the traversal can take. Thus, it is important to evaluate these aspects of the ontologies in order to assess the viability of our rewriting tool.

We propose the following metrics as a measure of complexity in this respect. The *Longest Path *is the maximum path length that may be computed within a given ontology. Each node in the path can be visited at most once so as to avoid looping. The longest path length provides an indication of the worse case for path calculation times. The *Average Paths per Journey *reflects the degree of path expansion within the rewriting algorithm, as each journey (e.g. from Node A to Node B) may have many different paths. The rewriting algorithm should be capable of returning all possible paths as each path may refer to a different expression of the query. When we consider that a single query may include multiple independent journeys, the possible query rewritings can become very large. The *Average Nodes per Path *is the average number of nodes that must be visited in order to return a single path. These metrics can affect the path calculation time as well as the complexity of the resulting query.

**Figure 10:**
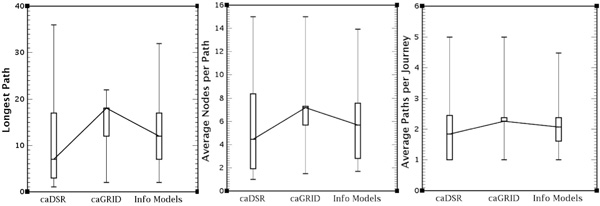
**The path metrics**. **Figure 10**: Three box plot diagrams showing path metrics for each subset of information models: caDSR, caGrid and Info Models. The path metrics considered are, from left to right: the longest path, the average number of nodes per path and the average number of paths per journey.

Figure 10 illustrates three box plots with the results of the path metrics for each project subset. We observe that while the longest path can have up to 36 nodes, for 75% of the projects in each category their length is less than 17 or 18. The median of the average path length varies between 4 and 7 nodes over the three subsets, and for 75% of the *Information Models *the average path length is less than 8. The median of the average paths is around 2 paths per journey, and for 75% of the projects in each category the average path per journey is less than 2.5. This indicates that we will be returning a low number of path combinations as a result. These results, then, verify that the paths within the ontologies are *manageable *and *appropriate *for our rewriting tool. By *appropriate*, we mean that, given that the tools are presented as part of a web-based interface with the time-restraints that accompany that implementation, results can be gathered within an appropriate time frame. By *manageable*, we suggest that the returned paths will not prove too complex for user interpretation. We also note that in all the metric diagrams, the caGrid subset is often very densely clustered around the mean. This is due to the fact that there are often many caGrid services for the same project that differ to one another very slightly or even not at all, which can result in multiple similar or identical results in our analysis.

### Ontology generation, module extraction and classification

**Figure 11:**
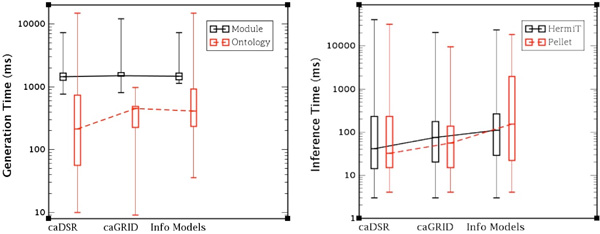
**Ontology and modules, generation and inference times**. **Figure 11**: The box plot diagram on the left shows the generation times for the NCIt module ontology and the annotated UML ontology for the three groups of information models (caDSR, caGrid and Info Models). The box plot diagram on the left depicts the inference times for the UML ontology using Hermit and Pellet reasoners. Both diagrams use logarithmic scale.

In order to isolate any overhead caused by variations in network performance, we extracted the XML corresponding to each project (or information model) in caDSR. This is a preliminary step so that the performance evaluation can be run locally, and we do not include any data or results of the performance of this stage. We generate four ontologies for each project: the NCIt module ontology (incorporating the concepts from NCIt relevant to the project), the annotated UML ontology (including the classes describing the UML model) and two inferred versions of the UML ontology. We generate the inferred ontologies by classifying the generated ontologies using both the HermiT and Pellet reasoners. We recorded the time for each generation and Figure 11 illustrates the times for the four ontologies of the each project grouped by subset. The times are presented in a logarithmic scale to enhance readability. We can see that the vast majority (75%) of NCIt modules take less than 2 seconds to generate and even less time for ontology generation. The classification of the generated ontologies is also timely, with the average inference of the Pellet and HermiT reasoners never longer than 100 milliseconds. We conclude that the generation and inference of the ontologies used in our approach does not present a barrier to the timely execution of the rewriting process.

### Query rewriting evaluation

**Figure 12:**
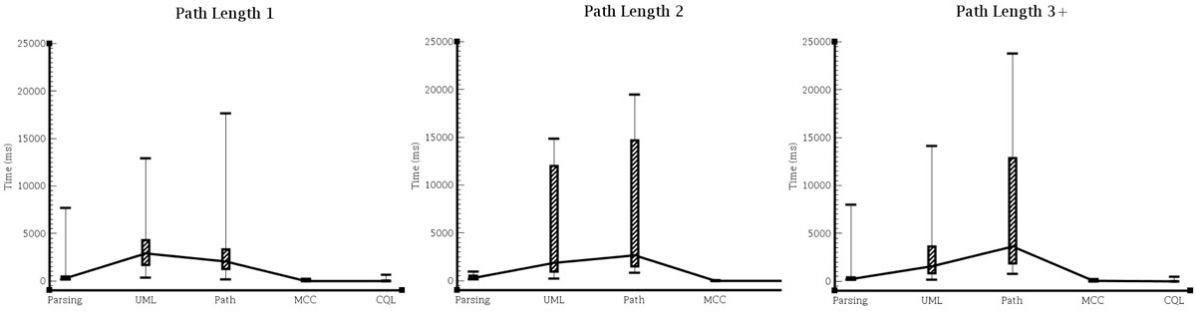
**Query rewriting performance**. **Figure 12**: Times taken in each stage of the query reformulation process (parsing, UML extraction, path finding, MCC conversion and CQL conversion) at varying path lengths.

We have developed a test suite of over one hundred queries of varying complexity in order to evaluate the query rewriting. More details on the performance evaluation can be found in the ConQueST website [37]. These queries are run over several services, which are publicly available from caGrid. The test suite currently queries the following models (available as services): caBio 4.2, caArray 2.4, caTissue 2.1 and PIR 1.2. The results are presented in Figure 12, which shows the times of each stage of the query reformulation process. These correspond to each stage of query rewriting: parsing, UML extraction, path finding, MCC conversion and CQL conversion. We grouped the test queries by query path length and these are presented in Figure 12. The path length refers to the number of intermediate nodes in the rewritten query. We can see from Figure 12 that, while the path length has an effect on the time taken at the path finding stage, the other stages of implementation remain largely unaffected. We therefore maintain that, given our analysis of paths within our target ontologies described above, we can provide query reformulation in a timely and efficient manner.

There are two principal factors that affect the performance of the path-finding stage of the query rewriting process; the length (complexity) of the returned path and the number of explanations requested to describe that path. The length of the returned path is the length (as an indication of complexity) of the path that is found between two nodes. We have shown in Figure 12 that there is a correlation between the length of the resulting path and the time taken in generation, although we accept that the overall effect is minimal. The path-finding stage makes use of an *explanation generator *[42] in order to find paths through the ontologies. We can ask for a number of explanations for one particular journey but we have no control over the order in which they are returned and we can make no inferences of how long each explanation took. Due to the black-box nature of the *explanation generator*, it is difficult to make any further assumptions of the internal processes at this stage. Rather, we endeavour to present a thorough evaluation of the performance of this stage to ensure the suitability of the method. During the rewriting evaluation described above, the path-finder was configured to return only a single explanation and, therefore, a single path for each query. The explanations returned during the path-finding process, while technically correct according to the ontology, are not necessarily desirable or biologically relevant. It is, therefore, sometimes necessary to request multiple explanations in order for the user to choose the desired path. The number of explanations requested has a marked influence of the time taken to return the paths.

**Figure 13:**
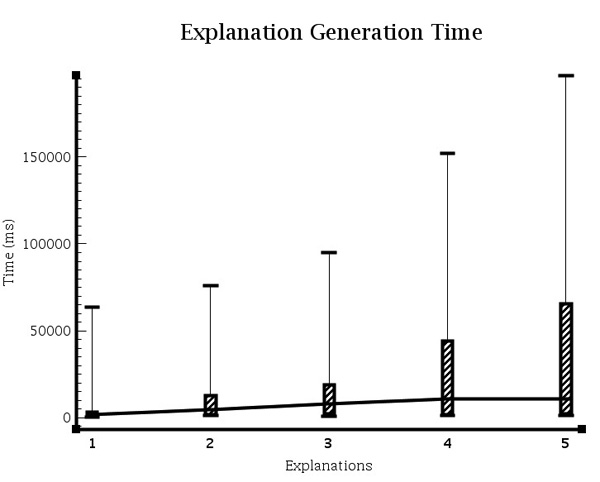
**Path finding performance**. **Figure 13**: Path-finding times for varying numbers of explanations, ranging from 1 to 5. Each explanation generates a path.

Figure 13 shows the time taken during the path-finding stage configured to return various numbers of explanations. Each requested explanation will result in the return of an additional single path. Looking at Figure 13, two things are clear. Firstly, as more explanations are requested, the time required for the path-finding stage increases. In some outlier cases, the path-finding times are very high (more than 3 minutes). Due to the the explanation generator being a black-box component using ontology reasoning, as we have already mentioned, it is difficult to assess the precise reasons behind these very long anomalies. Secondly, we can see that despite the lengthy times of some queries, the average time for a query remains relatively constant, with only a gentle correlation as we request more explanations. We therefore maintain, based on the average times, that query rewriting can be provided in a timely manner although care should be taken when requesting increased numbers of alternative paths.

## Conclusions

The realisation of personalised medicine requires the integration of data from a variety of scientific disciplines, such as molecular biology, pathology, radiology and clinical practice. Software infrastructures have been developed to facilitate the discovery and management of these types of data in oncology, including the NCRI ONIX system and the NCI caBIG^® ^infrastructure.

The caBIG^® ^infrastructure is based on the caGrid service-oriented middleware, which follows a federated Local-As-View approach to data integration by defining mappings from distributed data sources to a global-schema. The global-schema is realised by the NCI thesaurus ontology describing the cancer domain. The NCI thesaurus ontology is used to provide unambiguous meaning to the data sources. However, it is not currently used to provide a unified view for querying the data sources. Current querying capabilities in caGrid rely on the structure of the data sources.

This paper has presented an ontology-based querying system, which works over service-oriented and model-driven infrastructures for sharing cancer data. The design relied on generating ontologies from existing information models and reformulating ontology queries into resources' queries. The implementation was based on the caGrid infrastructure, but the approach could be used over similar model-driven software infrastructures. This work has extended our previous results [9] with the theory and implementation to handle federated queries, a more extensive evaluation of the query reformulation process, and the development of a graphical user interface aimed at cancer researchers. This paper has described the entire approach in detail, presenting:

*a*) the generation of customised OWL2 ontologies from annotated UML models, based on the ISO11179 standard for metadata registries. This differs from traditional UML-to-OWL conversions and it supports annotations with primary concept and qualifiers;

*b*) an analysis of the generated ontologies by determining several relevant ontology metrics, existing and new metrics that justify the viability of our rewriting technique;

*c*) an extended version of the query reformulation stages (including query rewriting and translation) to transform a domain ontology-based query into queries for a single resource or multiple resources; the latter involves the definition of join conditions, which can be found automatically by capitalising on the semantic annotations of the data sources; two simple use cases to illustrate the reformulation stages;

*d*) a caGrid analytical service implementing the OWL Generation facility;

*e*) an analysis of the capabilities of the caGrid query languages, both CQL and DCQL;

*f*) an extensive performance evaluation of the OWL generation, module extraction, querying rewriting and translation process.

## Methods

### OWL generation

UML is the *de-facto *visual modelling language for object-oriented design and the foundation for model-driven architectures. The ISO/IEC 11179 metadata registry in caGrid relies on UML class diagrams and their mappings to the NCIt ontology. In order to manage and reason about UML models and their annotations, we engineered OWL ontologies as a unified representation of the domain and data sources. In the following sections, we describe the approach for OWL generation, as outlined in the *Ontology-based Queries *section. The generation approach includes OWL models of UML class diagrams, OWL models of the NCIt-based annotations, and the extraction of modules from the NCIt ontology so that only the relevant concepts and properties for each data source are considered.

We observe that the generated ontologies contain only concepts and properties, i.e. terminological components or *TBoxes*. The assertion components, or *ABoxes*, correspond to the instances in the data sources.

#### OWL model of UML class diagrams

First, we present our customised UML-to-OWL transformation. This transformation differs from previous approaches transforming UML to OWL (for more details see [9,43]). We then describe the transformation and the use cases presented above to give examples.

Every UML element is related to its counterpart in the *UML model *ontology: all UML classes and attributes are defined as subclasses of *UMLClass *and *UMLAttribute*, respectively (see equations 1 and 2 below, where the prefixes are: *c*: for the caBIO ontology, *u*: for the UML model ontology, *n*: for the NCIt ontology and l: for the list ontology). We note that the name of an OWL class corresponding to an attribute includes the class name to avoid duplications and for associations, it includes its domain and range. All the UML associations are sub-properties of *hasAssociation *(equation 4), and the datatype property *hasValue *is used to specify the type of the attributes (equation 3) as an existential restriction. Contrary to other UML-to-OWL transformations, we represent UML attributes as OWL classes. This is required so that the ontology-based queries can include the concepts associated with attributes.

(1) c:Chromosome⊑u:UMLClass

(2) c:Chromosome_number⊑u:UMLAttribute

(3) c:Chromosome_number⊑∃u:hasValue.xsd:string

(4) c:Chromosome_locationCollection_Location⊑u:hasAssociation

UML subclass and superclass relationships are represented with subsumption (Eq. 5). For each UML class, existential restrictions are added for its associations (Eq. 6) and attributes (Eq. 7). While UML does not explicitly represent inherited associations, our OWL representation makes them explicit, modelling the semantics of UML. For example, as the UML class *Location *has an association *chromosome *with the class *Chromosome*, this association is inherited on the subclass *SNPPhysicalLocation *(Eq. 8).

(5) c:CytogeneticLocation⊑c:Location

(6) $$\begin{array}{ccc} \text{c:Chromosome} & ⊑ & \exists \text{c:Chromosome\_locationCollection\_Location}\text{.}\\ & & \text{c:Location} \end{array}$$

(7) c:Chromosome⊑∃u:hasAttribute.u:Chromosome_number

(8) $$\begin{array}{ccc} \text{c:SNPPhysicalLocation} & ⊑ & \exists \text{c:Location\_chromosome\_Chromosome}\text{.}\\ & & \text{c:Chromosome} \end{array}$$

We note that the generated OWL ontologies comply with OWL2EL [44], an OWL2 profile specifically designed to allow efficient reasoning of large terminologies, which is polynomial in the size of the ontology. While OWL2EL disallows universal quantification on properties, it does allow the inclusion of transitive properties. Thus, it is suitable for our UML-to-OWL transformation customised for the rewriting approach already outlined.

#### OWL representation of the semantic annotations

Apart from representing the UML model, we also model its mapping to NCIt, as maintained in caDSR. Through the CDEs, UML elements are annotated with a primary concept, which indicates the meaning of the element. In turn, a list of qualifier concepts may be used to modify the primary concept, providing a specific meaning [5]. As OWL2 does not natively supports the representation of lists, we used Drummond *et *al's design pattern for sequences [28] to model primary concepts and qualifier lists. The following equations give some examples of the modelling of the semantic annotations of UML classes (Eq. 9) and attributes (Eq. 10) with a single concept. Equation 11 models the class *cSNPPhysicalLocation *as a *n:Location *qualified with *l:Chromosome*_*Band *and *n:Single*_*Nucleotide*_*Polymorphism*.

(9) c:Chromosome⊑n:Chromosome

(10) c:Chromosome_numer⊑n:Name

(11) $$\begin{array}{ccc} \text{c:SNPPhysicalLocation} & ⊑ & \text{n:Location}⊓\text{(1:OWLList}⊓\\ & & \exists \text{1:hasContents}\text{.n:Chromosome}⊓\\ & & \exists \text{1:hasNext}\text{.(1:OWLList}⊓\\ & & \exists \text{1:hasContents}\text{.n:Single\_Nucleotide\_Polymorphism))} \end{array}$$

#### Module extraction from NCI thesaurus ontology

The NCIt ontology is very large, as it provides a common vocabulary for the whole cancer domain [7]. Each caGrid data service is, in general, concerned with data pertaining to more specific domains than the whole NCIt ontology. Thus, for each caGrid data service referring to a subset Σ of the NCIt vocabulary, there is a subset of terms and relationships from NCIt that is *relevant*, called a *module *from the ontology [45]. The module  represents all knowledge about the terms of the *signature *Σ. One of the approaches to *relevance *is logic-based: the module  is relevant for the terms Σ if all the consequences of the ontology that can be expressed over Σ are also consequences of M [45]. We follow that approach by Sattler *et al *[45] and extract an NCIt module for each of the information models in caGrid. For succinctness and efficiency, we use this module, as opposed to the whole NCIt ontology, for the semantic annotations of UML models and subsequent reasoning. We observe that we removed the disjoint axioms from the NCIt modules, as we noted before [43,46] that using subsumption to represent UML class to concept mapping may result in inconsistent ontologies as the annotations for a single class may come from two high-level branches in NCIt that are declared as disjoint.

### Query reformulation

**Figure 14:**
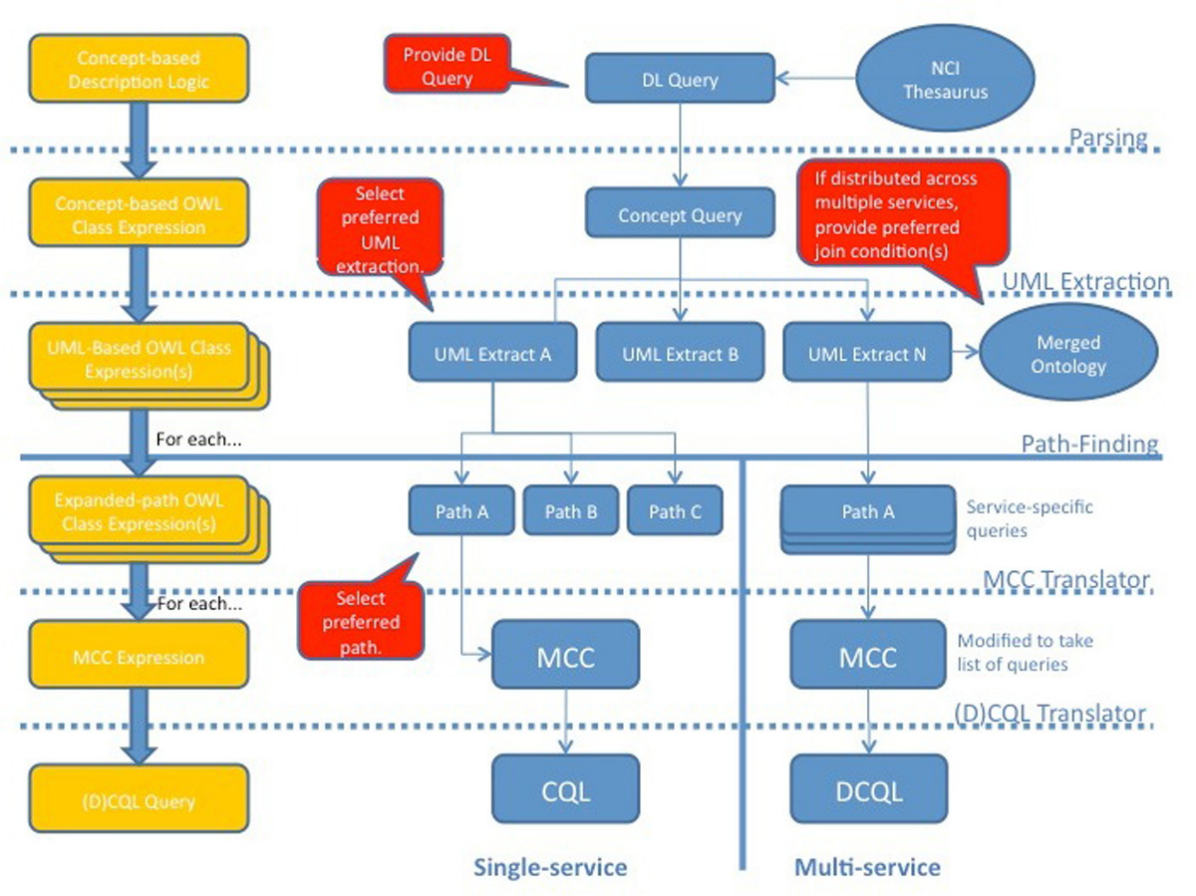
**Query reformulation stages**. **Figure 14**: The stages of query rewriting for both single and multiple target data services are depicted in blue. The form of the query at the different stages is represented in yellow and in red, we show the points of user interaction.

This section describes how an ontology-based query is rewritten and then translated, first to the intermediate optimisation language MCC and subsequently to the target CQL/DCQL languages. While the overall approach is similar to our previous work [9], we have comprehensively improved it, including extending the translation of queries over distributed data sources. In this section, we describe the query translation steps for both single and multiple-service queries. In most cases, the stages are the same (or negligibly different). We make clear the steps that are significantly different in the approach. We provide Figure 14 as an illustration of the query reformulation process. Within the figure, we make mention of the following;

• The constituent stages of the rewriting, describing the branching of the process for both single and multiple services (blue).

• The form of the query at each stage of the process (yellow).

• The points of user-interaction (red).

#### Parsing

First, the user query is syntactically parsed. The query uses concepts from the NCIt, the UML model ontology and the list ontologies [28]. If this stage fails, the user will be required to correct the query before continuing the reformulation process.

#### UML extraction

Initially, we express the query using NCIt concepts with the benefit that we require no knowledge of the structure of the underlying UML model and therefore, the query can be run over all the data services containing the concepts used. Having made this assertion, we must then translate the concepts to specific UML classes for specific data sources. This process is facilitated by our generated ontologies that specify a subclass relationship between a concept and the corresponding UML classes or UML attributes, depending on their position in the query. Therefore, in order to perform UML extraction, we must look to the relevant concept in the ontology and, using a reasoner, retrieve the subclasses of that concept that are also subclasses of the class UMLClass or of the class UMLAttribute, respectively. This denotes that the OWL class represents a UML class or UML attribute.

It is often the case that a single NCIt concept will correspond to many UML classes and, in such cases, each corresponding UML class is returned to form one single possible query. Therefore, the outcome of the UML extraction is a combination of possible queries given the extracted UML classes or attributes. Through the graphical interface, the user will be required to select the preferred UML extraction. In the second use case presented above, one possible UML extraction for the Concept-Based *Query 2 *for services caBIO and PIR is:

cabio:NucleicAcidSequence **and **(**hasAssociation some **(pir:Gene **and hasAttribute some **pir:Gene_name="BRCA1")) **and **(**hasAssociation some **(pir:Organism **and hasAttribute some **pir:Organism_scientificName="homo sapiens"))

#### Data values extraction

As the generated ontologies do not contain instances, the semantic validation of the query, expressed as an OWL class expression, must ignore the data expressions. This step extracts the data expressions, which will be reinserted later on.

In the *Query 2 *use case, this step results in:

cabio:NucleicAcidSequence **and hasAssociation some **pir:Gene **and hasAssociation some **pir:Organism

#### Semantic validation

We use a reasoner to check that the resulting query can be satisfied. If the query cannot be satisfied, subsequent reformulation of the query is halted.

#### Path finder

##### Single data source path finder

This step deals with the ontology corresponding to the UML model of data source (the semantic annotations do not need to be considered further) and aims to find the path of UML classes related through the transitive property *hasAssociation*^2^. The path finder rewrites the expression using non-transitive properties, corresponding to UML associations, by using an explanation generator [42] that retrieves the justification for two classes to be connected via the transitive property, and thus allowing to find the intermediate classes. The path finder may find more than one path between a set of nodes and, in such cases, will return each path as a combination of possible queries for user selection. In *Query 1*, the path finder stage retrieves:

cabio:SNP and **hasAssociation some **cabio:SNPPhysicalLocation **and hasAssociation some **(cabio:Chromosome **and hasAttribute some **(cabio:Chromosome_number))

##### Federated path finder

**Figure 15:**
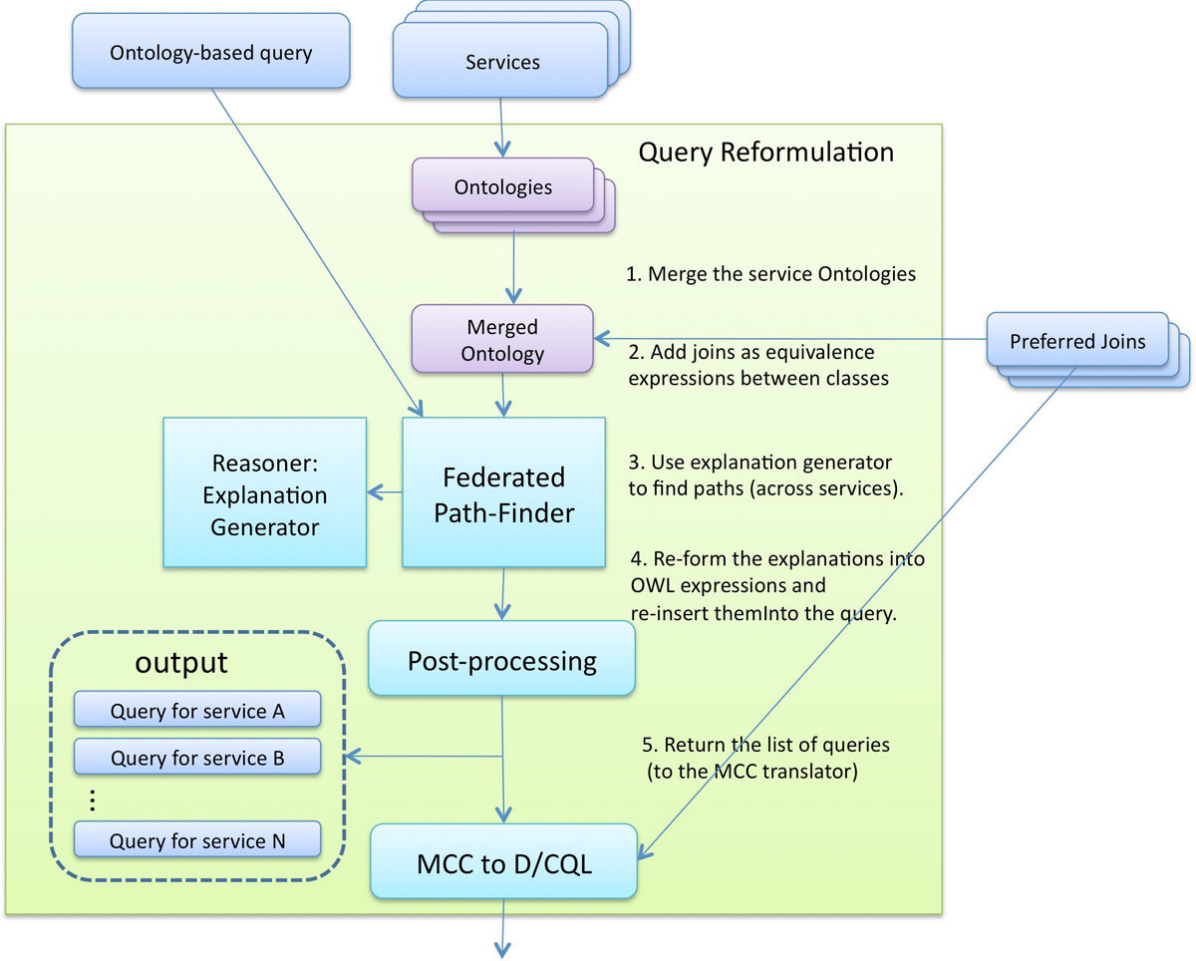
**Federated path finder**. **Figure 15**: Processes involved in finding paths in the information models when dealing with queries over multiple data services.

The process of query reformulation differs when translating to single-service CQL queries and multiple-service DCQL queries. Although the change is minimal or entirely absent in other stages, the path-finding stage has required the engineering of a new component, which we refer to as the *federated path finder*. Figure 15 illustrates the processes within the federated path finder, which again contain similarities to the single-service approach. The first step is to merge the UML model ontologies according to the classes present within the selected UML extraction. The merging of two ontologies results in an ontology which simply contains all the axioms of the two original ontologies.

At this point, we extract the join conditions of the ontologies within the single merged ontology. Given an scenario whereby we have no prior knowledge of where or how to join the ontologies, finding the join conditions between two ontologies is a two-part process. This process relies on the existing annotations with NCI thesaurus concepts. Firstly, we find the UML classes in each ontology that are annotated with the same concepts, implying that the classes are semantically equivalent. We then look at the attributes of those classes, searching for those that are also annotated with the same concepts. This provides us with the semantically equivalent attributes of the semantically equivalent classes. Through the interface, the user selects the preferred join conditions based on their biological relevance. An *Equivalent Class *axiom between the semantically-equivalent UML classes from the join condition is defined in the merged ontology. This new axiom allows to establish a path that can traverse from one ontology to another. Additionally, the join conditions are retained for lookup during the MCC translation. Additionally, we envision storing these join conditions for later use. These join conditions could be shared between users together with queries that use them.

The federated path-finder expands paths in the same way as the single-service path finder, except that explanations are generated from the merged ontology rather than a single ontology. This allows paths to be found that traverse more than one service (using the join conditions). When such an event occurs, we split the result each time we join to another ontology. The result of the federated path-finder, therefore, is a list of service-specific queries and the join conditions between them. This is subsequently passed to the MCC translator, which for federated queries has been extended to take a list of queries and, using the defined join conditions, forms the MCC expression.

In the *Query 2 *use case, two paths are found (one for each service):

Path finder result for caBIO

cabio:NucleicAcidSequence **and **(cabio:geneCollection **some **(cabio:Gene **and **cabio:proteinCollection **some **cabio:Protein))

Path finder result for PIR

pir:Protein **and **(pir:geneCollection **some **pir:Gene) **and **(pir:organismCollection **some **pir:Organism)

The join condition chosen between these two services includes the semantically equivalent classes *cabio:Protein *and *pir:Protein *with semantically equivalent attributes *cabio:uniProtCode *and *pir:uniprotkbEntryName*. This join condition links the two paths above.

#### Data values addition

At this point, we can retrieve the data expressions removed earlier and re-insert them into the corresponding OWL classes.

#### OWL expression to MCC translation

CQL and DCQL are object-oriented query languages, although no calculus or algebra has been defined for them. In order to provide a translation with D/CQL as target languages, we have decided to use the monoid comprehension calculus (MCC), as it is a formal framework to support object queries optimisations [25]. This formalism allows to manipulate object queries and, as we mentioned in the *Object-based Queries *section, using it as an intermediate language makes our approach general. Translating the ontology-based query to other target languages will involve only modifying the last step, MCC to C/DCQL, which is the only one dependent on caGrid. Also, adapting the system for future/modified versions of D/CQL will be simple. Additionally, the previous steps in the query reformulation process produce rewritings resulting from reasoning over the generated ontologies. This step, on the other hand, translates ontology-based expressions to monoid comprehensions, meaning that manipulation of the expressions is based on the calculus from now on. Last but not least, the use of MCC ensures support for optimisations.

Our approach is similar to the work by Peim *et al *[47], as they map description logics queries into the MCC. However, the are significant differences with our reformulation process. First, Peim *et al*'s solution is for GAV systems rather than LAV systems. In their system, each concept in the ontology is viewed as a named persistent set of database objects. Also, they assume that the data sources are described using the Object Definition Language (ODL). Moreover, while they use an expansion algorithm to rewrite an OWL expression based on a set of acyclic definitions, we follow the specific steps described in this section. We support query rewriting from OWL expression to the target languages using justifications of entailments [42] within the information model ontology.

The results of object queries are collections of homogeneous objects. The MC calculus offers a uniform notation for types representing collections, such as lists, bags and sets. The rationale is that the union operation over sets or bags, and the concatenation operation over lists are *monoid operations*. A monoid operation is associative and has an identity element. A monoid is an algebraic structure consisting of a set of elements and a monoid operation.

**Definition 1 (Monoid) ***A monoid is an algebraic structure defined by the triple *$<T_{⊕},⊕,Z_{⊕}>$, *where ** is a set*, ⊕ *is a binary associative operation *⊕: $⊕:T_{⊕}xT_{⊕}\to T_{⊕}$* called the *merge *function for the monoid, and the identity element *$Z_{⊕}$* is called the *zero *element for the monoid*.

The basic structure of the MCC is the monoid comprehension:

**Definition 2 (Monoid comprehension) ***A monoid comprehension is an expression of the form *$⊕\left\{e|\bar{q}\right\}$* where *⊕ *is a monoid operator called the *accumulator, *e is the *header *and *$\bar{q}=q_{1},...q_{n},n\geq 0$* is a sequence of *qualifiers. *A qualifier can take the form of a *generator, *v *← *e' with v a range variable and e' an expression constructing a collection, or a *filter *predicate*.

For each rewritten query after addition of data values, given as an OWL expression, we provide a transformation to MCC such that: the header variable is determined by the first concept in the query and the qualifiers are built for each of the remaining expressions. The header variable identifies the instances to be retrieved by the query, and the qualifiers specify the conditions that the instances must satisfy. The translation uses annotation properties included in the generated ontologies, which provide attributes such as *ClassName *for OWL classes representing UML classes, *AttributeName *for OWL classes representing UML attributes and *RoleName *for the name of the associations represented by object properties, which are sub-properties of *hasAssociation*.

Next, we define the reformulation function ℝ to translate OWL class expressions into MCC. The definition of ℝ is compositional: it is applied to the whole OWL class expression representing the query after UML extraction and data values addition, and subsequently to sub-expressions. Finally, the translation of sub-expressions is composed to produce the MCC expression that represents the overall translation. In the following definitions, *Expr*_*i *_represents a general OWL class expression, *A *and *B *represent OWL classes, C represents a constant and p represents an object property. The function ℝ_*var *_denotes the assignment of variables, such that ℝ_*var*_() creates a new variable, and ℝ_*var*_(*A*) retrieves the variable assigned to the OWL class *A *if it exists, otherwise it creates a new variable for *A*. If A is an OWL class representing a UML attribute, the function ℂ(A) retrieves the UML class containing the attribute A. The function D(p) retrieves the domain of the object property *p*.

(12) $$\mathbb{R}\left(Expr_{1}\;\text{and}\;Expr_{2}\right)=\left\{\begin{array}{cc} ⊎\left\{{\mathbb{R}}_{var}\left(\right)|\mathbb{R}\left(A\right)=C\right\}, & \\ \text{if}\;Expr_{1}=\text{hasAttribute some A and }Expr_{2}=p_{2}\text{value}\;C & \\ ⊎\{{\mathbb{R}}_{var}\left(\right)|\mathbb{R}\left(Expr_{1}\right)\;\text{and}\;\mathbb{R}\left(Expr_{2}\right), & \text{otherwise} \end{array}\right)$$

(13) $$\mathbb{R}\left(A\right)=\left\{\begin{array}{cc} ⊎\left\{{\mathbb{R}}_{var}\left(A\right)|{\mathbb{R}}_{var}\left(A\right)\leftarrow \mathbb{R}\left(A\right)\right\} & \text{A represents a UML class}\\ {\mathbb{R}}_{var}\left(\mathbb{C}\left(A\right)\right).\text{AttributeName}\left(A\right) & \text{A represents a UML attribute} \end{array}\right)$$

(14) $$\mathbb{R}\left(\text{p}\;\text{some}\;Expr\right)=\left\{\begin{array}{c} ⊎\left\{newVar={\mathbb{R}}_{var}\left(\right)|newVar\leftarrow {\mathbb{R}}_{var}\left(D\left(p\right)\right).\text{RoleName}\left(p\right),newVar\leftarrow \mathbb{R}\left(Expr\right)\right\}\\ \text{if p represents a sub-property of hasAssociation}\\ \mathbb{R}\left(Expr\right),\text{ if p represents the object property hasAttribute} \end{array}\right)$$

(15) $$\mathbb{R}\left(Expr_{1}\;\text{or}\;Expr_{2}\right)=\text{or}\;\left\{{\mathbb{R}}_{var}\left(\right)|\mathbb{R}\left(Expr_{1}\right),\mathbb{R}\left(Expr_{2}\right)\right\}$$

When receiving a list of OWL class expressions from the previous step (federated path finder with data values reinserted) and the join conditions, the MCC generator uses the reformulation function above for each of the OWL class expressions. It then combines them into a single MCC expression by defining the join condition as *v*_*i*_.*localAttributeName *= *v*_*j*_. *foreignAttributeName*, where *v*_*i *_and *v*_*j *_correspond to the local and foreign semantically-equivalent classes, respectively.

Once the algorithm obtains an MCC expression, it is normalised using the rules described in [25] and simplified, i.e. the number of variables used is reduced.

In the *Query 2 *use case, the resulting MCC expression is:

(16) $$\begin{array}{c} ⊎\{v_{0}|\\ \begin{array}{c} \begin{array}{c} \;\;\;\;v_{0}\leftarrow \text{gov}\text{.nih}\text{.nci}\text{.cabio}\text{.domain}\text{.NucleicAcidSequence,}\\ v_{1}\leftarrow v_{0}\cdot \text{geneCollection,}\\ v_{\text{1}}\leftarrow \text{gov}\text{.nih}\text{.nci}\text{.cabio}\text{.domain}\text{.Gene,}\\ v_{2}\leftarrow v_{1}\cdot \text{proteinCollection,}\\ v_{2}\leftarrow \text{gov}\text{.nih}\text{.nci,cabio}\text{.domain}\text{.Protein}\\ v_{2}.uniProtCode=v_{3.}uniprotkbEntryName\\ v_{3}\leftarrow \text{edu}\text{.georgetown}\text{.pir}\text{.domain}\text{.Protein},\\ v_{4}\leftarrow v_{3}.\text{geneCollection,}\\ v_{4}\leftarrow \text{edu}\text{.georgetown}\text{.pir}\text{.domain}\text{.Gene,}\\ v_{4}.name{=}^{\prime \prime }\text{BRCA}1^{\prime \prime }\\ v_{5}\leftarrow v_{3}.\text{organismCollection,}\\ v_{5}\leftarrow \text{edu}\text{.georgetown}\text{.pir}\text{.domain}\text{.Organism}\\ v_{5}.scientificName{=}^{\prime \prime }\text{homo sapiens”\}} \end{array} \end{array} \end{array}$$

#### MMC to D/CQL translation

Translating the MCC expression into CQL includes the following; define as *Target *the type of the variable that appears in the header; including an *Association *per each pair of generators, one determining the name (the class to which they belong) and the other identifying the role name; include an *Attribute *restriction for each filter.

When the MCC expression contains a sub-expression corresponding to a join condition, the result will be a DCQL query. Each MCC expression is translated similarly to the description above, where the overall target is the *TargetObject*. Additionally, the expressions of the join conditions are used to define the *ForeignAssociation(s)*, where the equivalent attributes are used to define the *JoinCondition *and the target from the second MCC expression is the *ForeignObject*.

## Competing interests

The authors declare that they have no competing interests.

## Authors' contributions

AGB designed the approach for ontology-based queries over cancer data, which included two components: one for the generation of ontologies from information models and another one for the query reformulation process. The latter component used the Monoid Comprehension Calculus (MCC) as intermediate language, and AGB provided the translation rules from OWL class expressions to MCC. AGB and BT implemented the both components and designed the evaluation methods. BT compiled the results of the evaluation. BT wrapped the ontology generation code as a caGrid service and developed the graphical user interface to expose the query system. AF provided vision, scope, requirements analysis throughout the project. All authors participated in revision and have read and approved the manuscript.

## Foot Note

^1^Several videos demonstrating the interface can be found at http://www.cs.ucl.ac.uk/staff/a.gonzalezbeltran/conquest/

^2^We note that the ontology is compliant with the OWL2 EL profile, as OWL2 EL supports the use of transitive object properties. For more information, see http://www.w3.org/TR/owl2-profiles/
